# Types I, II, V, and VI secretion system genes in clinical uropathogenic *Escherichia coli* associate with antibiotic susceptibility, biofilm formation, and persister cells

**DOI:** 10.1128/spectrum.00286-26

**Published:** 2026-07-15

**Authors:** Paulina Soból, Weronika Kęsek, Maja Kirzyc, Edyta Podsiadły, Maciej Obrębski, Weronika Skowrońska, Jakub P. Piwowarski, Marcin Równicki

**Affiliations:** 1 Student Scientific Association “Herbarium”, Department of Pharmaceutical Biology, Faculty of Pharmacy, Medical University of Warsaw 417849, Warsaw, Poland; 2 Department of Pharmaceutical Microbiology and Bioanalysis, Faculty of Pharmacy, Medical University of Warsaw 417849, Warsaw, Poland; 3 Microbiota Lab, Department of Pharmaceutical Microbiology and Bioanalysis, Faculty of Pharmacy, Medical University of Warsaw 417849, Warsaw, Poland; 4 Laboratory of Microbiology, University Center for Laboratory Medicine, Medical University of Warsaw 417849, Warsaw, Poland; 5 Department of Dental Microbiology, Faculty of Medicine and Dentistry, Medical University of Warsaw 417849, Warsaw, Poland; 6 Department of Pharmaceutical Biology, Faculty of Pharmacy, Medical University of Warsaw 417849, Warsaw, Poland; UCI Health, Orange, California, USA

**Keywords:** UPEC, secretion systems, biofilms, *Escherichia coli*, persister cells, antibiotic resistance

## Abstract

Uropathogenic *Escherichia coli* (UPEC) often causes recurrent urinary tract infections, where biofilms and persister cells can promote recalcitrance. This study evaluated whether secretion-system marker genes associate with antimicrobial susceptibility, biofilm formation, and biofilm-associated persistence in clinical urinary isolates. A total of 71 isolates were screened by PCR for *fimH* and markers of secretion systems: type I (*hlyA*), type II (*gspD*), type V (*ag43*), and type VI (*hcp*). Minimum inhibitory concentrations (MICs) were measured by Etest, biofilm biomass by crystal violet assay, and persister survival in strong biofilm producers after antibiotic challenge. *fimH* was detected in 91.5% isolates; *gspD* and *ag43* were common (71.8% and 80.3%), while *hlyA* and *hcp* occurred in 32.4% and 46.5%. Marker co-occurrence was frequent, with 19.7% carrying all four secretion markers, and 8.5% with no secretion markers detected. A total of 81.7% isolates formed biofilm, including 18.3% strong producers. Individual marker prevalence did not differ across biofilm categories. Carriage of *hlyA* and *hcp* was associated with lower MIC distributions for selected antibiotics (including fosfomycin, ciprofloxacin, and ampicillin-sulbactam), whereas *gspD* and *ag43* showed no consistent MIC associations. The biofilm category was linked to non-susceptibility to ampicillin-sulbactam, but not the other agents tested. Strong biofilm producers showed biphasic killing consistent with persister formation, and *hcp* carriage tended to align with higher ciprofloxacin survival. These findings map secretion-system markers in clinical UPEC and suggest that a type VI secretion system may track with biofilm-associated persistence.

**IMPORTANCE:**

Recurrent urinary tract infections (rUTI) often relapse after antibiotic treatment because bacteria can generate persister cells—temporary survivors that tolerate antibiotics without being genetically resistant. Clinicians, therefore, lack markers that flag isolates likely to persist. In 71 clinical urinary *Escherichia coli* isolates, this study surveyed genes encoding several secretion systems, molecular machines that help bacteria interact with each other and with host tissues. These markers were widespread and frequently co-occurred, but they did not track with stronger biofilm formation. However, a type VI secretion system marker (*hcp*) was associated with higher survival during ciprofloxacin exposure in strong-biofilm isolates, linking virulence-associated machinery with biofilm-associated persistence. Genetic signatures that reflect persistence, rather than routine resistance, could help prioritize follow-up testing and support development of strategies that target hard-to-eradicate rUTI infections.

## INTRODUCTION

Urinary tract infections (UTIs) are among the most common bacterial infections worldwide and impose a substantial clinical and economic burden (1, 2). In elderly patients, UTIs are reported frequently and are associated with prolonged hospitalization and complications (3). A major clinical challenge is recurrence: approximately one quarter of women experience symptomatic recurrence within 12 months after an initial episode, highlighting recurrent UTI (rUTI) as a distinct disease state shaped by both host and bacterial determinants (4).

Uropathogenic *Escherichia coli* (UPEC) is the predominant cause of community-acquired UTIs and remains a leading agent of both uncomplicated and complicated infections (4). Treatment is often initiated empirically, frequently before urine culture and susceptibility results are available (5). Although this enables rapid symptom control, repeated antimicrobial exposure can select for multidrug-resistant strains and contribute to therapeutic failure (6). However, antimicrobial resistance alone does not fully explain rUTI recalcitrance, as recurrence is frequently caused by the same strain, consistent with bacterial persistence despite apparently appropriate therapy (7).

Biofilm growth and persister cell formation are two complementary mechanisms that can promote persistence independent of inherited resistance (8, 9). In biofilms, UPEC forms structured communities embedded in an extracellular matrix that can reduce antibiotic efficacy through restricted drug penetration, altered local physiology, and nutrient or oxygen gradients that enrich slow-growing subpopulations (10, 11). Persister cells are transient phenotypic variants that survive antibiotic exposure through reversible metabolic downshifts rather than genetic resistance (12). After treatment, persisters can resuscitate and repopulate, contributing to relapse with the same strain (13). In UPEC, persistence has also been linked to intracellular lifestyles, including intracellular bacterial communities and quiescent reservoirs that may seed recurrence after antimicrobial withdrawal (14, 15). The substantial heterogeneity of biofilm formation and persister frequency among clinical UPEC isolates suggests that strain-specific genetic features may contribute to persistence-relevant phenotypes (16).

UPEC persistence depends on coordinated virulence functions that promote adhesion, invasion, tissue damage, nutrient acquisition, and competition within polymicrobial communities (17). Type I fimbriae, particularly the FimH adhesin encoded by *fimH*, mediate urothelial attachment and invasion and are strongly associated with the initiation of infection (18). In addition, UPEC genomes commonly encode multiple secretion systems that export toxins and effector proteins and may influence biofilm maturation and persistence (19). The present study focused on marker genes linked to types I, II, V, and VI secretion pathways: *hlyA*, *gspD*, *ag43*, and *hcp*. These targets were selected based on their prevalence in clinical UPEC and reported links to virulence or biofilm-associated phenotypes. *hlyA* (T1SS) encodes α-hemolysin, which contributes to epithelial damage and immune modulation (20), whereas *gspD* (T2SS) and ag43 (T5SS autotransporter) have been implicated in biofilm maturation through secretion of extracellular factors and autoaggregation, respectively (21–23). *hcp*, a T6SS marker, is associated with contact-dependent interbacterial competition that can shape biofilm community structure (24). In contrast, type III secretion systems are rarely detected in clinical UPEC and are more characteristic of other *E. coli* pathotypes, while type IV secretion systems are incompletely defined in UPEC and lack a robust single-gene marker suitable for broad PCR screening (25, 26). We therefore focused on these four marker genes as the most biologically supported targets for association testing in a clinical isolate collection.

Although secretion systems can influence biofilm architecture and microbial interactions, direct evidence linking carriage of T1SS, T2SS, T5SS, and T6SS marker genes to persister-like survival in clinical UPEC isolates remains limited. To address this gap, we tested whether variation in selected adhesin and secretion-system marker loci is associated with phenotypic heterogeneity relevant to UPEC persistence. We screened *fimH*, *hlyA*, *gspD*, *ag43*, and *hcp* in a collection of Polish clinical urinary *E. coli* isolates and assessed their relationships with *in vitro* biofilm formation, antimicrobial susceptibility profiles, and biofilm-associated persister-like survival. Defining these genotype-phenotype associations may help identify bacterial features linked to persistence-relevant traits and support future studies on risk stratification and targeted therapeutic approaches for difficult-to-eradicate UPEC infections.

## RESULTS

### Isolate collection and prevalence of adhesin and secretion-system marker genes

We analyzed 71 clinical UPEC isolates and three reference strains (CFT073, 536, and *E. coli* K-12 MG1655). Clinical isolates were recovered from routine urine cultures collected at two hospitals affiliated with the Medical University of Warsaw (Warsaw, Poland) and submitted for clinically suspected UTI; isolates were classified as UPEC based on the clinical indication and urinary source.

As an initial UPEC marker, we first assessed *fimH*, which encodes the type I fimbrial adhesin and is central to uroepithelial attachment and early surface colonization. *fimH* was detected in 65/71 clinical UPEC isolates (91.5%), and in all three control strains (Table 1). We then screened secretion-system marker genes and detected *hlyA* (hemolysin A; T1SS-secreted toxin) in 23/71 strains (32.4%), *gspD* (T2SS core component) in 51/71 strains (71.8%), *ag43* (type V autotransporter) in 57/71 strains (80.3%), and *hcp* (T6SS marker) in 33/71 strains (46.5%; Table 1).

**TABLE 1:** Prevalence of *fimH* and secretion system-associated genes among UPEC clinical isolates (*n* = 71) and the reference strains

Gene	Clinical (*n* = 71)	CFT073	UPEC 536	K-12 MG1655
*fimH*	65 (91.5%)	+	+	+
*hlyA*	23 (32.4%)	+	+	−
*gspD*	51 (71.8%)	+	+	−
*ag43*	57 (80.3%)	+	+	+
*hcp*	33 (46.5%)	+	−	−

Additionally, we analyzed the distribution of secretion system-associated gene combinations across the UPEC clinical isolates (Table 2). Single-marker carriage was observed for T2 (4/71; 5.6%) and T5 (9/71; 12.7%), whereas T1 and T6 were not detected alone. The most common exact profile was carriage of all four secretion system markers (14/71; 19.7%), followed by T2 + T5 + T6 (12/71; 16.9%) and T2 + T5 (10/71; 14.1%). Within the mutually exclusive profiles, T2 + T5 alone was detected in 10/71 isolates (14.1%; Table 2). However, when co-detection was analyzed in a non-mutually exclusive manner, T2 and T5 markers were present together in 43/71 isolates (60.6%), including isolates that also carried T1 and/or T6 markers. In contrast, six isolates (8.5%) were *fimH* positive but negative for all secretion markers (*hlyA*, *gspD*, *ag43*, and *hcp*; Table 2). A detailed per-isolate summary of *fimH* detection and marker-gene patterns is provided in the Supplementary Materials (Table S1).

**TABLE 2:** Distribution of secretion system marker-gene co-occurrence among UPEC isolates^*a*^

Co-occurrence	Detection	Number of strains *n* (%)
No secretion markers detected	*fimH*	6 (8.5%)
1	T2	4 (5.6%)
	T5	9 (12.7%)
2	T1 + T5	2 (2.8%)
	T2 + T5	10 (14.1%)
	T2 + T6	4 (5.6%)
	T5 + T6	3 (4.2%)
3	T1 + T2 + T5	7 (9.9%)
	T2 + T5 + T6	12 (16.9%)
4	T1 + T2 + T5 + T6	14 (19.7%)

^*a*^ Percentages are calculated using *n* = 71 UPEC clinical isolates. T1/T2/T5/T6 correspond to detection of markers *hlyA*,* gspD*,* ag43*, and *hcp*, respectively. Detection rates are calculated as the proportion of *n* = 71 clinical isolates carrying each specific gene combination and are mutually exclusive.

### Biofilm formation in UPEC

**Fig 1:**
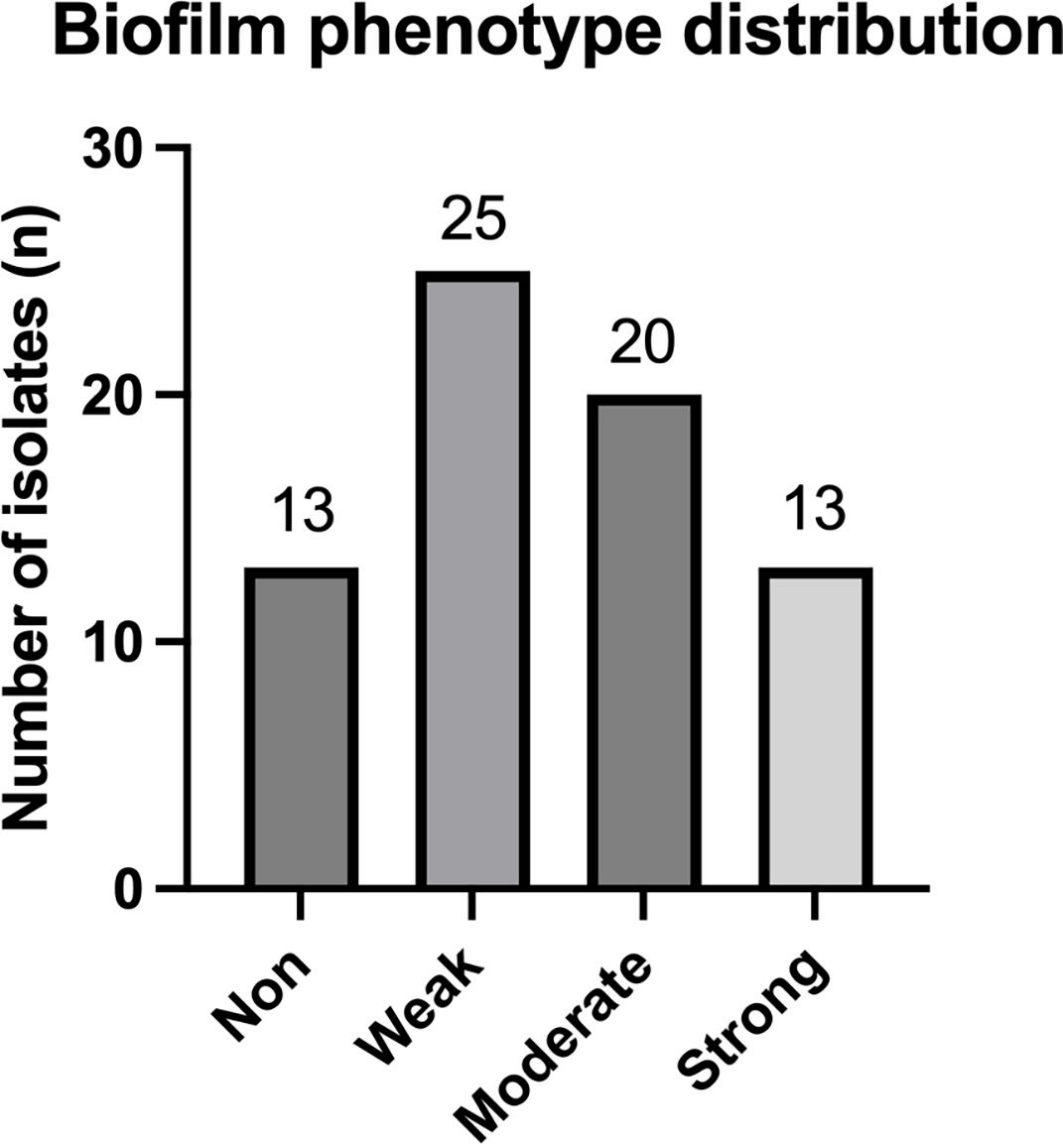
Biofilm phenotype distribution among UPEC clinical isolates. UPEC clinical isolates (*n* = 71) were categorized as non-biofilm producers (13; 18.3%), weak biofilm producers (25; 35.2%), moderate biofilm producers (20; 28.2%), or strong biofilm producers (13; 18.3%).

We next performed phenotypic characterization of the clinical isolates by assessing biofilm formation and antibiotic susceptibility. UPEC clinical isolates were assigned to four biofilm categories according to the biofilm biomass produced: non-biofilm producers (13/71; 18.3%), weak biofilm producers (25/71; 35.2%), moderate biofilm producers (20/71; 28.2%), and strong biofilm producers (13/71; 18.3%; Fig. 1). The control strains displayed distinct biofilm phenotypes: *E. coli* K-12 MG1655 was a non-biofilm producer, UPEC 536 was a moderate biofilm producer, and CFT073 was a strong biofilm producer. Detailed data on the biofilm levels for each individual strain are provided in Table S1.

### *fimH* and secretion-system marker gene distribution by biofilm phenotype

When grouped by biofilm phenotype, *fimH* and secretion system-associated gene prevalence varied across categories (Table 3). *fimH* was highly prevalent across all phenotypes (84.6%–100%). The prevalence of secretion system markers showed modest numerical variation, with *hcp* increasing from non-biofilm producers (30.7%) to strong biofilm producers (53.8%), *ag43* lowest among strong biofilm producers (69.2%) relative to weak (84%) and moderate (80%) producers, *hlyA* ranging from 20.0% to 53.8%, and *gspD* remaining consistently common (64.0%–76.9%); however, none of these differences were statistically significant (*χ*² tests, all *P* ≥ 0.109; Table 3). Across the full set of UPEC clinical isolates, the most frequent co-occurrence pattern was *gspD + ag43* (43/71; 60.6%), followed by *gspD + hcp* (30/71; 42.3%) and *ag43 + hcp* (29/71; 40.8%; Table S2). Patterns were generally distributed similarly across biofilm phenotypes. The *gspD + hcp* profile (T2SS + T6-associated markers) showed a nominal distributional difference across categories in the unadjusted analysis (*χ*² =7.93, *P* = 0.048, Table S2); however, this association did not remain significant after Benjamini-Hochberg correction for multiple testing with a false discovery rate (FDR)-adjusted *q* value of 0.528(Table S2). Therefore, this observation should be interpreted as exploratory. All other secretion-system marker combinations showed no significant association with biofilm phenotype after correction for multiple testing (*χ*² tests, *P* ≥ 0.100; Table S2).

**TABLE 3:** Distribution of *fimH* and secretion system-associated genes across biofilm categories

Gene	Non-biofilm (*n* = 13)	Weak (*n* = 25)	Moderate (*n* = 20)	Strong (*n* = 13)	*χ*²	*P* value
*fimH*	13/13 (100%)	22/25 (88%)	19/20 (95%)	11/13 (84.6%)	2.723	0.436
*hlyA*	7/13 (53.8%)	6/25 (24%)	4/20 (20%)	6/13 (46.2%)	6.063	0.109
*gspD*	10/13 (76.9%)	16/25 (64%)	15/20 (75%)	10/13 (76.9%)	1.190	0.755
*ag43*	11/13 (84.6%)	21/25 (84%)	16/20 (80%)	9/13 (69.2%)	1.376	0.711
*hcp*	4/13 (30.7%)	12/25 (48%)	10/20 (50%)	7/13 (53.8%)	1.696	0.638

### Secretion-system markers and antibiotic susceptibility

**Fig 2:**
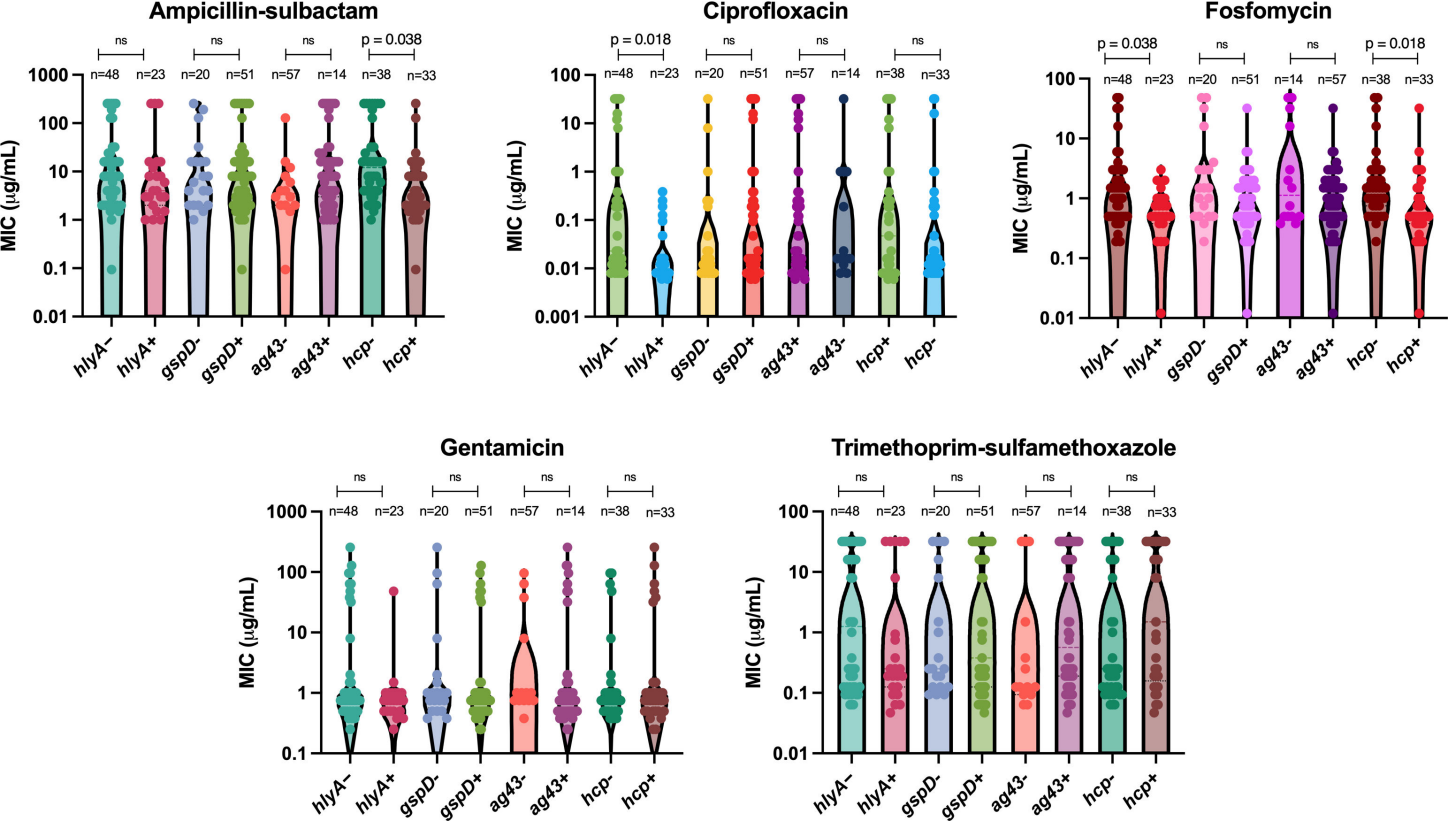
Secretion-system markers associate with MIC distributions for tested antibiotics. Violin plots show MIC (µg/mL) distributions for clinical UPEC isolates grouped by carriage of each specific secretion-system marker gene: *hlyA*, *gspD*, *ag43*, and *hcp*. Each dot represents a single isolate; central lines indicate the median and interquartile range, and *n* stands for the number of isolates. Pairwise comparisons were performed using Mann-Whitney *U* tests with Benjamini-Hochberg FDR correction. Adjusted *P* values are shown on the graph for statistically significant comparisons; ns indicates non-significant comparisons.

Antimicrobial susceptibility was determined by Etest (representative images shown in Fig. S1), and results were interpreted according to CLSI breakpoints. The UPEC isolates displayed variable susceptibility to the antimicrobials tested (Table S1). Fosfomycin demonstrated the highest efficacy, with 100% classified as susceptible. Conversely, ampicillin-sulbactam and trimethoprim-sulfamethoxazole exhibited the lowest susceptibility rates, at 60.6% (43/71) and 62.0% (44/71), respectively. Gentamicin and ciprofloxacin demonstrated intermediate susceptibility, affecting 84.5% (60/71) and 78.9% (56/71) of isolates tested, respectively. Detailed minimum inhibitory concentration (MIC) values for each strain and each antibiotic are presented in Table S1. To assess whether secretion-system markers tracked with antimicrobial susceptibility, MIC distributions were compared between marker-positive and marker-negative clinical isolates. Neither *gspD* (T2SS) nor *ag43* (T5SS) showed a consistent association with MIC values across the antibiotics tested. In contrast, *hlyA*-positive isolates showed lower ciprofloxacin MICs than *hlyA*-negative isolates, with median MICs of 0.012 µg/mL and 0.023 µg/mL, respectively (adjusted *P* = 0.018). *hlyA*-positive isolates also showed lower fosfomycin MICs than *hlyA*-negative isolates, with median MICs of 0.5 µg/mL and 1.5 µg/mL, respectively (adjusted *P* = 0.038). Similarly, *hcp*-positive isolates showed lower fosfomycin MICs than *hcp*-negative isolates, with median MICs of 0.5 µg/mL and 1.5 µg/mL, respectively (adjusted *P* = 0.018), and lower ampicillin-sulbactam MICs, with median MICs of 4 µg/mL and 12 µg/mL, respectively (adjusted *P* = 0.038; Fig. 2 and Table S3). Overall, carriage of selected secretion system markers, particularly *hlyA* and *hcp*, was associated with lower MIC values, whereas these genetic determinants showed no correlation with elevated antimicrobial resistance in this isolate collection.

### Biofilm formation is associated with ampicillin-sulbactam resistance

We next compared antibiotic susceptibility across biofilm categories (Table 4). Overall, ampicillin-sulbactam resistance differed by biofilm category (*χ*², *P* = 0.031), with the highest resistance rates among non-biofilm and weak producers (53.8% and 56.0%, respectively) and lower rates among moderate and strong biofilm producers (25.0% and 15.4%; Table 4). In contrast, for the other antimicrobials tested, resistance frequencies tended to show only gradual shifts across biofilm categories and did not reach statistical significance. Gentamicin and ciprofloxacin resistance both displayed a stepwise increase from non-biofilm to strong biofilm producers, while trimethoprim-sulfamethoxazole resistance fluctuated across groups without a clear monotonic pattern. All isolates remained susceptible to fosfomycin (Table 4).

**TABLE 4:** Antimicrobial resistance across biofilm formation categories^*a*^

Antibiotic	Non-biofilm *n* = 13	Weak *n* = 25	Moderate *n* = 20	Strong *n* = 13	*χ* ^2^	*P* value
Fosfomycin	0/13 (0.0%)	0/25 (0.0%)	0/20 (0.0%)	0/13 (0.0%)	NA	
Gentamicin	1/13 (7.7%)	3/25 (12.0%)	3/20 (15.0%)	4/13 (30.8%)	3.158	0.368
Ciprofloxacin	2/13 (15.4%)	4/25 (16.0%)	5/20 (25.0%)	4/13 (30.8%)	1.557	0.669
Ampicillin-sulbactam	7/13 (53.8%)	14/25 (56.0%)	5/20 (25.0%)	2/13 (15.4%)	8.896	0.031
Trimethoprim-sulfamethoxazole	4/13 (30.8%)	13/25 (52.0%)	5/20 (25.0%)	5/13 (38.5%)	3.803	0.284

^*a*^ Resistance was defined using CLSI M100 34th edition susceptible MIC cutoffs. NA stands for not applicable because all isolates were classified as fosfomycin susceptible (MIC ≤64 µg/mL).

### Persister cell formation in strong biofilm producers

**Fig 3:**
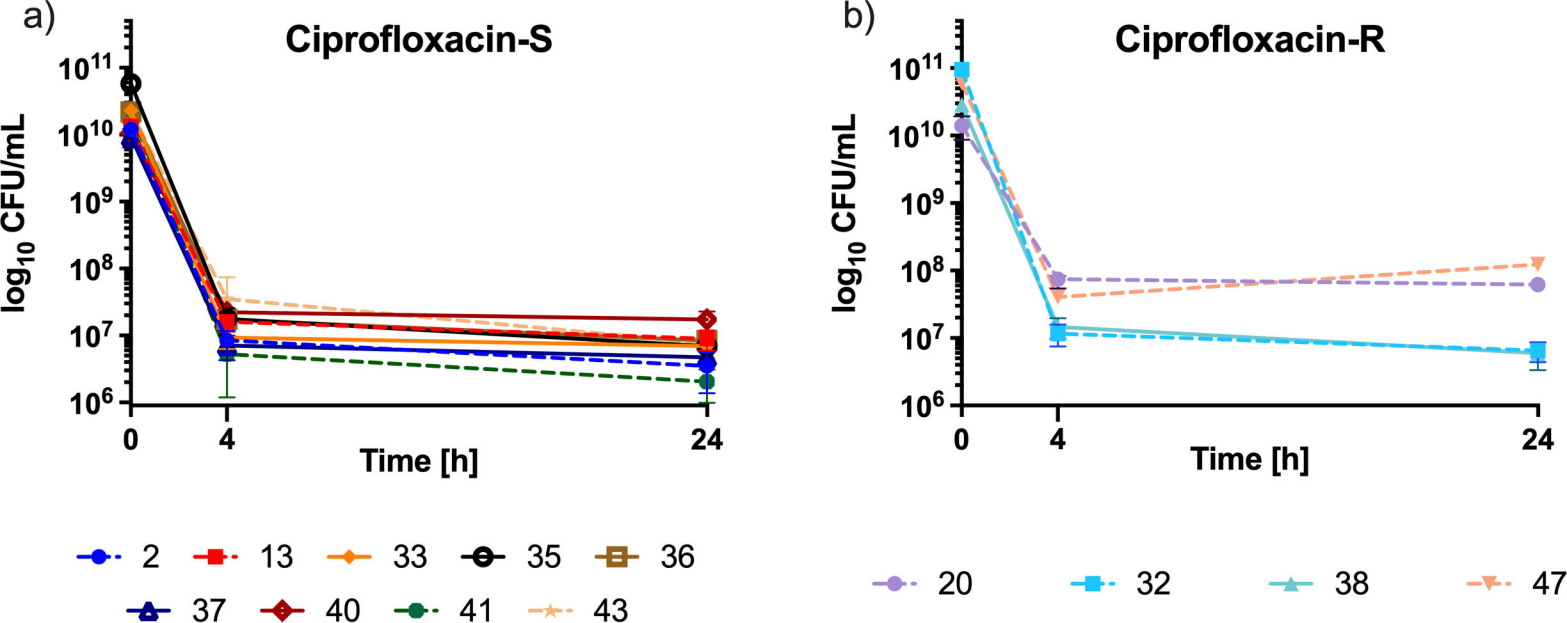
Persister assay survival dynamics of strong biofilm-forming UPEC isolates during ciprofloxacin challenge. Viable counts are shown as log_10_ CFU/mL at 0, 4, and 24 h after antibiotic exposure. (**a**) Ciprofloxacin-susceptible isolates. (**b**) Ciprofloxacin-resistant isolates. Each line represents an individual isolate; dashed lines indicate *hcp*-positive isolates, and solid lines indicate *hcp*-negative isolates. Points denote CFU/mL at each time point, and error bars (when visible) indicate mean ± SD (*n* = 3).

Since biofilm growth is associated with enrichment of antibiotic-tolerant persister cells, we quantified persister formation in 13 strong biofilm-forming clinical isolates. Ciprofloxacin (5 μg/mL) was used as a clinically relevant agent for UTI therapy, and gentamicin (200 μg/mL) was used for persister assays in ciprofloxacin-resistant isolates. Ciprofloxacin-susceptible isolates demonstrated rapid killing, with an approximately 3–4 log₁₀ reduction in colony-forming unit (CFU) per milliliter within 4 h, followed by a survival plateau through 24 h, consistent with the presence of a persister subpopulation (Fig. 3a). Among ciprofloxacin-resistant isolates, the reduction in viable counts was heterogeneous: two strains, isolate 20 and isolate 47, showed limited ciprofloxacin-mediated reduction, whereas the remaining two ciprofloxacin-resistant isolates displayed a reduction-and-plateau pattern comparable to ciprofloxacin-susceptible isolates (Fig. 3b). Together, these data indicate that ciprofloxacin MIC alone does not predict the magnitude of the persister-associated survival plateau. To further characterize the ciprofloxacin-resistant isolates, we also assessed their survival following gentamicin exposure, which differentiated strains with marked killing from those with minimal reduction, consistent with variable aminoglycoside susceptibility (Fig. S2).

### Secretion-marker patterns among strong biofilm producers

When persister levels were compared across secretion-system profiles, *hcp*-positive isolates, including isolates 2, 13, 20, 32, 41, 43, and 47, showed a descriptive shift toward higher 24 h survival values after ciprofloxacin challenge (Fig. 3 and Table S1). Specifically, 24 h ciprofloxacin survival ranged from 2.98 × 10⁶ to 1.23 × 10⁸ CFU/mL in *hcp*-positive isolates, compared with 6.13 × 10⁶ to 1.73 × 10⁷ CFU/mL in *hcp*-negative isolates (Fig. 3 and Table S1). This identifies *hcp* as a candidate marker for enhanced persistence in strong biofilm-forming UPEC; however, this observation is based on a limited number of isolates and needs further validation in expanded cohorts and functional studies. In contrast, the presence of *hlyA*, *gspD*, and/or *ag43* showed no clear association with consistent differences in persister levels for either of the antibiotics tested.

## DISCUSSION

Recurrent UTIs are frequently driven by the ability of pathogens to survive and persist within the urinary tract despite host immune responses and apparently appropriate antimicrobial therapy (27). In this study, we screened four secretion system-associated marker genes representing type I (*hlyA*), type II (*gspD*), type V (*ag43*), and type VI (*hcp*), as well as *fimH*, in 71 clinical UPEC isolates. We then examined their association with biofilm formation, antimicrobial susceptibility, and persister cell frequency—key determinants of rUTI (28). Three key findings appeared. First, secretion-marker genes were widespread and commonly co-occurred across isolates. Second, gene prevalence showed no strong relationship with biofilm category overall. Third, among strong biofilm producers, *hcp*-positive isolates showed descriptively higher 24 h ciprofloxacin survival values than *hcp*-negative isolates, consistent with a potential link between a T6SS and biofilm-associated persistence.

### *fimH* is maintained regardless of biofilm capacity

An important finding was the consistency of *fimH* prevalence across all biofilm categories, suggesting that *fimH* is maintained regardless of biofilm-forming capacity. This reinforces the concept that type I fimbriae represent a fundamental fitness determinant for UPEC survival and persistence in the urinary tract and is not a biofilm-specific adaptation that would be enriched only among high-biofilm phenotypes (29). Interestingly, within the small subset of strong-biofilm producers that were *fimH*-negative, two of three strains were *ag43* positive. Although this observation is strictly descriptive due to the limited number of isolates, it is consistent with the idea that *ag43*-mediated aggregation and surface-associated growth could partially compensate for the loss or absence of type I fimbriae in some genetic backgrounds (22, 30). This potential “substitution” model should be tested in larger cohorts and with functional assays, but it highlights how urinary isolates may reach a strong biofilm phenotype via alternative adhesion/aggregation strategies.

### Secretion markers are common and frequently co-occur

The high prevalence of *gspD* (T2SS), *ag43* (T5SS), and *hcp* (T6SS), together with frequent multi-gene combinations—with ~20% of isolates carrying all four markers (*hlyA*, *gspD*, *ag43*, and *hcp*)—demonstrates that secretion-system-associated loci are common in clinical UPEC (31–33). Their frequent co-occurrence suggests that these genes are often physically or functionally linked, possibly through shared genomic islands or coordinated selective pressures (34, 35). Notably, single-marker carriage was observed (T2- and T5-only profiles), but most marker-positive isolates carried multiple markers. This co-occurrence may reflect the organization of UPEC virulence-associated loci within larger mobile or pathogenicity-associated genomic regions. For example, the PAI-CFT073-pheV pathogenicity island contains hemolysin-associated genes and ag43 among other fitness and virulence determinants, illustrating how such loci may be co-inherited in clinical UPEC lineages (34–36). Reference UPEC strains provide useful models for studying secretion-system biology (37), although they do not capture the full genetic diversity of clinical urinary isolates, as illustrated by the variable marker-gene combinations observed in our collection. A striking pattern was that T1 (*hlyA*) was never detected alone: every T1-positive isolate also carried T5 (*ag43*), suggesting a nonrandom association between these loci in this collection. In contrast, the finding that six isolates have no secretion system markers is intriguing and warrants investigation: these strains may rely on alternative virulence mechanisms not captured by our gene panel, may represent recent mutational losses, or may be experiencing fitness costs that limit their prevalence in clinical settings.

### Secretion system markers show limited association with bulk biofilm biomass

A key finding is that the prevalence of individual secretion-system markers (*hlyA*, *gspD*, *ag43*, and *hcp*) did not significantly differ across biofilm phenotype categories. This was somewhat unexpected, given that several secretion systems have been implicated in biofilm formation in laboratory settings. For instance, T1SS-secreted hemolysin has been proposed to damage the urothelium and promote inflammatory responses that may facilitate persistence (20, 38, 39); T5SS has been shown to promote auto-aggregation and community formation (21, 22, 40); and T6SS systems can participate in interbacterial interactions within biofilm environments (41, 42). However, biofilm formation is multifactorial and depends on adhesion, motility, matrix production, metabolism, stress responses, and regulatory networks rather than any single determinant (43); therefore, we also examined combined marker profiles. At the level of combined profiles, the *gspD + hcp* pattern (T2SS + T6-associated markers) showed only a nominal distributional difference across biofilm categories in the unadjusted analysis and did not remain significant after correction for multiple testing. Thus, this finding should be interpreted as exploratory. Future studies using larger, phylogenetically resolved isolate collections will be needed to determine whether joint carriage of T2SS- and T6SS-associated features reflects lineage structure, regulatory states, or functional contributions to biofilm formation.

### ag43 shows an inverse relationship with biofilm strength

Notably, ag43 prevalence showed an inverse relationship with biofilm strength, being highest among non-biofilm producers (84.6%) and lowest among strong biofilm producers (69.2%). This pattern suggests that T5SS-mediated autoagglutination, despite being an adhesion-related mechanism, may not be essential for robust biofilm formation in clinical UPEC (21). One plausible interpretation is that strong biofilm producers may rely more on fimbriae-based or exopolysaccharide-mediated aggregation pathways, making Ag43 less beneficial (44). Alternatively, maintaining Ag43 expression under biofilm conditions may impose fitness costs that outweigh its advantages (45, 46). While mechanistic explanations require targeted testing, the inverse association observed here emphasizes that “adhesion genes” do not always map linearly onto “stronger biofilm” phenotypes in clinical isolates.

### Type I and type VI secretion markers associate with antimicrobial susceptibility

We also explored whether secretion-marker carriage associates with MIC-defined antimicrobial resistance. We observed that *hlyA* and *hcp* carriage were associated with lower MIC values for selected antibiotics. Specifically, *hlyA*-positive isolates showed significantly lower ciprofloxacin and fosfomycin MICs, while *hcp*-positive isolates displayed lower fosfomycin and ampicillin-sulbactam MICs. This finding supports the view that virulence-associated marker carriage and antimicrobial resistance can be partially uncoupled and should be evaluated empirically rather than assumed to covary (47, 48). It also shows why we need to test these links in clinical isolates, instead of assuming they are the same as in lab strains. We also observed that biofilm phenotype was linked to ampicillin-sulbactam resistance frequency, with resistance highest among non-biofilm and weak producers (53.8% and 56.0%) and lower in moderate and strong producers (25.0% and 15.4%). While biofilms can reduce antibiotic efficacy through physiological tolerance mechanisms, we would expect an impact on other antimicrobials tested. However, the lack of significant association between biofilm category and susceptibility to gentamicin, ciprofloxacin, fosfomycin, or trimethoprim-sulfamethoxazole suggests that biofilm phenotype is not a strong universal predictor of resistance across drug classes. This finding supports the multifactorial nature of UPEC persistence—biofilm formation and planktonic MIC-defined resistance are distinct phenotypes that may be controlled by different genetic and environmental factors, and MIC assays do not imply biofilm formation under the *in vitro* conditions tested.

### Persister cell formation associates with type VI secretion marker

Among strong biofilm producers, *hcp* carriage trended toward higher persister-like survival during ciprofloxacin and/or gentamicin challenge. Although persister measurements were available for a limited number of strong biofilm isolates, this finding suggests *hcp* as a candidate marker of enhanced biofilm-associated persistence. However, this observation should be interpreted as hypothesis generating because this study assessed *hcp* carriage rather than *hcp* expression. Our findings align with literature data showing that T6SS-mediated interbacterial antagonism can generate spatial heterogeneity in laboratory models, with resistant or tolerant subpopulations arising in protected niches (49, 50). Our observation adds a clinical dimension to this phenomenon and suggests that, if expressed and functional, T6SS could actively shape biofilm architecture or interbacterial interactions in ways that promote persister enrichment. Notably, neither *hlyA*, *gspD*, nor *ag43* showed a clear association with persister levels, suggesting the specificity of the T6SS system in this context. This selectivity is important: it suggests that not all secretion systems equally contribute to persistence, and that a mechanistic understanding of individual secretion system roles may be required for targeted therapeutic approaches. In matched no-antibiotic controls, viable counts remained stable over the same time course (Fig. S3).

### Clinical and translational implications

Our findings have several implications for understanding rUTI pathogenesis and for developing risk-stratification approaches. First, although secretion-system markers were common among clinical UPEC, their associations were phenotype specific (e.g., low MICs for *hlyA* and *hcp*; high persistence for *hcp*), suggesting that simple gene-panel screening may not predict overall virulence or treatment response without considering genetic background and functional validation. Second, the lack of relationship between marker prevalence and bulk biofilm biomass suggests that these genes alone will not reliably predict biofilm formation. However, the association between *hcp* and persister survival suggests that T6SS markers may be useful biomarkers for identifying strains at higher risk of producing persistence phenotypes and causing recurrent infection. If validated in larger cohorts and prospective clinical studies, measurement of *hcp* in urine isolates could support risk stratification and motivate consideration of persistence-targeted interventions (e.g., higher-dose or prolonged antimicrobial therapy, combination therapy, and anti-biofilm agents). Third, our findings underscore that resistance and tolerance are distinct challenges: susceptible strains carrying *hcp* still form persisters and survive high-dose ciprofloxacin exposure despite low MIC values. This supports diagnostic strategies that complement both conventional susceptibility testing with assays capturing tolerance (e.g., biofilm-associated survival or persister readouts) to better characterize the pathogenic potential of UPEC isolates and inform personalized treatment strategies.

### Limitations

This study assessed the presence of secretion-system marker genes and did not measure their expression or activity (e.g., by qPCR); therefore, the observed associations do not establish functional contribution to virulence or persistence, and targeted validation will be needed. Additionally, the phylogenetic structure of isolates was not evaluated; future studies incorporating MLST or whole-genome sequencing may reveal whether secretion system gene profiles are associated with specific UPEC lineages. Finally, clinical metadata (e.g., recurrence patterns, specimen characteristics, treatment course, and patient outcomes) were not collected; prospective studies linking bacterial genotypes and phenotypes to rUTI outcomes would strengthen the translational relevance of this work.

## MATERIALS AND METHODS

### Bacterial strains and isolation

A total of 71 clinical UPEC isolates were recovered from urine specimens collected from symptomatic hospitalized patients at hospitals affiliated with the Medical University of Warsaw (Warsaw, Poland) and submitted for clinically suspected UTI. One strain per patient was included in the study. Initial isolation was performed on Columbia agar (Thermo Scientific, USA) at 35°C–37°C for 18–24 h. Colonies presumptively identified as *E. coli* were screened on chromogenic selective media (CHROMagar Orientation, CHROMagar, France) or MacConkey agar (Thermo Scientific, USA) at 35°C–37°C for 18–24 h. Colonies displaying characteristic morphology—dark pink to reddish coloration on CHROMagar Orientation or MacConkey agar—consistent with lactose-fermenting gram-negative rods were confirmed by standard biochemical tests, including oxidase-negative and indole-positive reactions. Such colonies were classified as *E. coli* and selected for further molecular characterization. Throughout the study, we used three reference strains as controls: *E. coli* CFT073 (O6:K2:H1) (51) as the prototypical UPEC reference strain, UPEC strain 536 (O6:K15:H31) (17) as an alternative virulent lineage to validate that secretion system markers are detected across genetically distinct clinical UPEC isolates, and a non-pathogenic control *E. coli* K-12 MG1655 (52).

### Genomic DNA extraction

Genomic DNA was extracted from bacterial cell cultures according to the manufacturer’s protocol using the Invitrogen PureLink Genomic DNA Mini Kit (Thermo Fisher Scientific, USA). Briefly, overnight cultures of clinical isolates were centrifuged at 10,000 × *g* for 3 min, and the cell pellet was resuspended in Genomic Digestion Buffer. The lysate was treated with proteinase K and incubated at 55°C for 1 h. After incubation, RNase A was added to the lysate, vortexed, and incubated at room temperature for 2 min. Genomic Lysis Binding Buffer and 99% ethanol were added before purification. Genomic DNA was subsequently purified by silica spin columns and eluted in 50 μL of Elution Buffer. DNA concentration and purity were assessed using a NanoDrop spectrophotometer (Thermo Fisher Scientific, USA) by measuring absorbance at 260 nm and 280 nm; DNA samples with A260/A280 ratios between 1.8 and 1.9 were considered adequate for downstream PCR applications. A total of 74 bacterial strains were analyzed, comprising 71 clinical isolates and 3 control strains.

### Molecular characterization and PCR-based identification: detection of *fimH*

UPEC isolates were presumptively classified based on clinical criteria, and the *fimH* gene (encoding the type I fimbrial adhesin) was screened as a UPEC-associated marker by PCR amplification and agarose gel electrophoresis. PCR reactions were performed in a total volume of 25 μL containing the following components (Vazyme Biotech, China): 16.75 μL of nuclease-free water, 2.5 μL of Taq Buffer with MgCl_2_ (10 mM), 0.5 μL of dNTP Mix (200 μM each), and 0.25 μL of Taq DNA Polymerase (5 U/μL); primers (Genomed S.A., Poland): 1 μL of forward primer *fimH* (10 μM), 1 μL of reverse primer *fimH* (10 μM), and 3 μL of template genomic DNA (primer sequences and expected amplicon size are provided in Table S4). PCR amplification was performed using a C1000 Thermal Cycler (Bio-Rad Laboratories) under the following conditions: initial denaturation at 95°C for 3 min, followed by 34 cycles of denaturation at 95°C for 15 s, annealing at 55°C for 15 s, and extension at 72°C for 40 s, with a final extension at 72°C for 5 min and indefinite storage at 4°C. Positive and negative controls (no-template controls) were included in each PCR run.

### Detection of secretion-system marker genes

To identify secretion system-associated marker genes, we performed a multiplex PCR targeting *hlyA*, *gspD*, and *ag43*, and a separate PCR assay targeting *hcp* (details below). Primer sequences and expected amplicon sizes are provided in Table S4.

### Detection of hlyA, gspD, and ag43 (T1SS, T2SS, and T5SS marker genes)

Multiplex PCR reactions targeting *hlyA*, *gspD*, and *ag43* (T1, T2, and T5SS marker genes, respectively) were performed in a total volume of 25 μL containing: 12.75 μL of nuclease-free water, 2.5 μL of Taq Buffer with MgCl_2_ (10 mM), 0.5 μL of dNTP Mix (200 μM each), 1 μL each of forward and reverse primers for *hlyA* (10 μM), 1 μL each of forward and reverse primers for *gspD* (10 μM), 1 μL each of forward and reverse primers for *ag43* (10 μM), 0.25 μL of Taq DNA Polymerase (5 U/μL), and 3 μL of template genomic DNA. Thermal cycling conditions were as follows: an initial denaturation at 95°C for 3 min, followed by 34 cycles of denaturation at 95°C for 20 s, annealing at 55°C for 15 s, and extension at 72°C for 1 min, with a final extension at 72°C for 5 min and indefinite storage at 4°C.

### Detection of hcp (T6SS core gene)

PCR reactions for the *hcp* gene (T6SS) were performed in a total volume of 25 μL containing: 16.75 μL of nuclease-free water, 2.5 μL of Taq Buffer with MgCl₂ (10 mM), 0.5 μL of dNTP Mix (200 μM each), 1 μL of forward primer *hcp* (10 μM), 1 μL of reverse primer *hcp* (10 μM), 0.25 μL of Taq DNA Polymerase (5 U/μL), and 3 μL of template genomic DNA. Thermal cycling conditions were as follows: initial denaturation at 95°C for 3 min, followed by 34 cycles of denaturation at 95°C for 15 s, annealing at 53°C for 15 s, and extension at 72°C for 30 s, with a final extension at 72°C for 5 min and indefinite storage at 4°C.

### Agarose gel electrophoresis

PCR products were separated by agarose gel electrophoresis to visualize the presence of target genes. For *fimH* and *hcp* amplicons, 1% agarose gel (wt/vol) in 1× TAE buffer (40 mM Tris, 20 mM acetate, 1 mM EDTA, pH 8.0) was used. For multiplex PCR products, 1.5% agarose gel (wt/vol) in 1× TAE buffer (40 mM Tris, 20 mM acetate, 1 mM EDTA, pH 8.0) was used. Both gel preparations were supplemented with SimplySafe nucleic acid stain (EURx, Poland) according to the manufacturer’s protocol. Electrophoresis was conducted at 100 V for 45 min (fimH and hcp gels) or 90 min (multiplex PCR gel). GeneRuler 1 kb Plus DNA Ladder or GeneRuler 1 kb DNA Ladder (Thermo Fisher Scientific, USA) was used as a molecular weight marker. PCR products were visualized using G:BOX (Syngene, UK), and the presence of amplicons was determined by comparing observed band sizes to the expected amplicon sizes (Fig. S4).

### Antibiotic susceptibility

Antimicrobial susceptibility to fosfomycin, gentamicin, ciprofloxacin, ampicillin-sulbactam, and trimethoprim-sulfamethoxazole was determined using Etest strips (bioMérieux, France; Liofilchem, Italy; Biomaxima, Poland). Bacterial suspensions adjusted to 0.5 McFarland units were inoculated onto Mueller-Hinton II agar (Becton Dickinson BBL, USA) using sterile cotton swabs. Etest strips were placed on the dried agar surface and incubated at 37°C for 24 h. MICs were determined by reading the intersection point where bacterial growth inhibition met the gradient on the strip. MICs were interpreted according to CLSI M100 breakpoints (34th Edition, 2024): fosfomycin ≤64 µg/mL (S), gentamicin ≤2 µg/mL (S), ciprofloxacin ≤0.25 µg/mL (S), ampicillin-sulbactam ≤8/4 µg/mL (S), and trimethoprim-sulfamethoxazole ≤2/38 µg/mL (S) (53). Quality control was performed using *E. coli* ATCC 25922. Isolates were classified as susceptible (S) or resistant (R) based on CLSI breakpoints, and the prevalence of each category was calculated as a percentage of the 71 clinical UPEC isolates tested.

### Biofilm formation assay

Overnight cultures of tested UPEC strains were prepared in 3 mL of brain heart infusion broth with shaking at 140 RPM at 37°C. On the following day, 20 µL of overnight culture was inoculated into sterile 96-well microtiter plates (flat bottom) containing 180 µL of Luria-Bertani (LB) broth per well (final inoculum approximately 1% [vol/vol]). Plates were incubated statically at 37°C for 24 h to allow biofilm formation. Following incubation, the supernatant was removed without disturbing the biofilm, and wells were washed twice with phosphate-buffered saline (PBS). Biofilms were fixed with 99% methanol and incubated for 15 min at room temperature. Then, methanol was removed, and plates were air-dried completely at room temperature. Fixed biofilms were stained with 0.1% crystal violet solution for 20 min at room temperature. Excess stain was removed by rinsing three times with distilled water, and plates were air-dried at room temperature. Crystal violet was eluted with 33% acetic acid and incubated for 20 min at room temperature. A minimum of 100 µL of the eluted biofilm suspension from each well was transferred to a clean 96-well plate, and absorbance was measured at 590 nm using a microplate reader (BioTek PowerWave XS2, Biotek, USA).

Biofilm formation was classified relative to the non-biofilm control strain *E. coli* K-12 MG1655 according to the following categories: OD ≤ OD control (non-biofilm producer), OD > OD control to 2 × OD control (weak biofilm producer), OD > 2 × OD control to 4 × OD control (moderate biofilm producer), and OD > 4 × OD control (strong biofilm producer). This classification scheme is based on optical density thresholds relative to the K-12 control strain (54, 55). Experiments were performed in at least three independent biological replicates (*n* = 3).

### Persister cells in biofilm

To assess persister cell survival within biofilms, the initial biofilm cultivation was performed identically to the biofilm formation assay described above, up to the PBS washing step. Subsequent antibiotic exposure, biofilm disruption, and CFU recovery steps were performed according to a previously published biofilm-persister protocol, with minor modifications (56). Briefly, 120 µL of LB containing ciprofloxacin (final concentration, 5 µg/mL) was added to each well, corresponding to ≥20× the highest MIC among ciprofloxacin-susceptible strong biofilm producers, and plates were incubated for an additional 24 h at 37°C. Ciprofloxacin-resistant isolates were assessed in a separate assay using gentamicin (200 µg/mL) as an alternative antibiotic challenge. To disrupt the biofilm and harvest surviving cells, plates were vortexed at 900 RPM for 5 min, followed by 5 min of sonication in a water bath. This detachment step was used to improve recovery of biofilm-embedded cells and reduce underestimation of surviving CFUs caused by incomplete biofilm disruption or bacterial aggregation. The same vortexing and sonication conditions were applied consistently to all samples and time points. The resulting suspension was homogenized by pipetting and transferred to sterile microcentrifuge tubes. The pooled suspensions were centrifuged at 6,000 RPM for 3 min, and the pellets were resuspended in PBS. Serial 10-fold dilutions were prepared in sterile 96-well plates. Surviving CFUs were quantified by spotting 10 µL of each dilution in triplicate onto LB agar plates. Plates were then incubated for 24 h at 37°C, and colony-forming units were enumerated to quantify surviving (persister) cells. CFU counts of persisters were performed at 0, 4, and 24 h following antibiotic addition. The presence of a surviving subpopulation following this high-dose antibiotic treatment indicated a characteristic biphasic killing curve, consistent with persister-like survival dynamics. Matched no-antibiotic biofilm controls were included for each strain and processed in parallel using the same biofilm disruption and CFU enumeration procedure. Experiments were performed in at least three independent biological replicates (*n* = 3).

### Statistical analysis

All statistical analyses were performed in GraphPad Prism (GraphPad Software; version 10.3, San Diego, CA, USA). Unless otherwise stated, tests were two-tailed, and *P* < 0.05 was considered statistically significant. Associations between gene carriage (present/absent for *hlyA*, *gspD*, *ag43*, and *hcp*) and biofilm phenotype (non/weak/moderate/strong) were evaluated using Pearson’s *χ*^2^ test of independence. MIC distributions or persister burdens between gene-positive and gene-negative groups were performed using the Mann-Whitney test with Benjamini**-**Hochberg FDR correction for multiple comparisons applied separately for each antibiotic.

## Data Availability

The data supporting the findings of this study are available in the Zenodo repository at https://doi.org/10.5281/zenodo.20330854.

## SUPPLEMENTAL MATERIAL

Supplemental figures and tables spectrum.00286-26-s0001.pdf Additional experimental details.

ASM does not own the copyrights to Supplemental Material that may be linked to, or accessed through, an article. The authors have granted ASM a non-exclusive, world-wide license to publish the Supplemental Material files. Please contact the corresponding author directly for reuse.
