# Supplementary material for: Types I, II, V, and VI secretion system genes in clinical uropathogenic Escherichia coli associate with antibiotic susceptibility, biofilm formation, and persister cells
Source: Microbiol Spectr. 2026 Jul 15;14(8):e00286-26. doi: 10.1128/spectrum.00286-26 (PMC13436075; doi:10.1128/spectrum.00286-26)
Supplement: Supplementary file 1 [file spectrum.00286-26-s0001.pdf]

Supplemental Material:

Type I, II, V, and VI Secretion System Genes in Clinical Uropathogenic *Escherichia coli* Associate with Antibiotic Susceptibility, Biofilm Formation, and Persister Cells

Paulina Soból[a,b], Weronika Kęsek[a,b], Maja Kirzyc[c], Edyta Podsiadły[d,e], Maciej Obrębski[c], Weronika Skowrońska[f], Jakub P. Piwowarski[c], Marcin Równicki[c#]

[a] Student Scientific Association "Herbarium", Department of Pharmaceutical Biology, Faculty of Pharmacy, Medical University of Warsaw, Warsaw, Poland

[b] Department of Pharmaceutical Microbiology and Bioanalysis, Faculty of Pharmacy, Medical University of Warsaw, Warsaw, Poland

[c] MicrobiotaLab, Department of Pharmaceutical Microbiology and Bioanalysis, Faculty of Pharmacy, Medical University of Warsaw, Warsaw, Poland

[d] Laboratory of Microbiology, University Centre for Laboratory Medicine, Medical University of Warsaw, Warsaw, Poland

[e] Department of Dental Microbiology, Medical University of Warsaw, Warsaw, Poland

[f] Department of Pharmaceutical Biology, Faculty of Pharmacy, Medical University of Warsaw, Warsaw, Poland

Running Head: Secretion systems and persistence in clinical UPEC

#Address correspondence to Marcin Równicki, marcin.rownicki@wum.edu.pl

Paulina Soból and Weronika Kęsek contributed equally to this work.

Table S1 Summary of phenotypic and genotypic characterization of all tested strains. For each strain, mean biofilm biomass (average OD) and the corresponding biofilm category are shown alongside antibiotic susceptibility expressed as minimum inhibitory concentrations (MICs). MIC values were interpreted according to CLSI M100, 34th edition, 2024 breakpoints. Susceptible MICs are indicated in green and resistant MICs in red. The presence/absence of *fimH* and secretion system-associated genes is reported based on PCR assays. For strong biofilm producers, persister levels are provided as CFU/mL following antibiotic challenge. Control strains are marked with asterisks. Antibiotic abbreviations: FO, fosfomycin; GM, gentamicin; CIP, ciprofloxacin; SAM, ampicillin-sulbactam; TMP-SMX, trimethoprim-sulfamethoxazole.

		Biofilm		Antibiotics sensitivity (MIC)					PCR					Persisters in biofilm				
**Strain no.**	**Clinical reference no.**	**Average OD**	**Category**	**FO**	**GM**	**CIP**	**SAM**	**TMP SMX**	***fimH***	***hlyA* T1SS**	***gspD* T2SS**	***ag43* T5SS**	***hcp* T6SS**	**0h**	**CIP 4h**	**CIP 24h**	**GM 4h**	**GM 24h**
**11**	5544m25	0.152	Non-biofilm producer	0.38	0.75	0.008	3	>32	1	1	1	1	1					
**12**	5560m25	0.221	Non-biofilm producer	0.5	1	0.008	1	0.047	1	1	1	1	1					
**14**	m338328	0.171	Non-biofilm producer	0.5	0.5	≤ 0.25	1	≤ 8	1	1	1	1	1					
**30**	m/3550	0.225	Non-biofilm producer	1	0.75	0.016	1.5	0.19	1	1	1	1	1					
**50**	m/3632	0.222	Non-biofilm producer	2	0.75	0.016	>256	0.19	1	1	1	1	0					
**51**	6092m25	0.234	Non-biofilm producer	1	1	0.012	>256	0.125	1	1	1	1	0					
**52**	6172m25	0.216	Non-biofilm producer	1.5	1	0.008	>256	0.19	1	0	1	1	0					
**53**	6069m25	0.207	Non-biofilm producer	1	0.75	16	>256	>16	1	0	1	1	0					
**55**	m/3803	0.196	Non-biofilm producer	16	8	>1	4	0.25	1	0	0	0	0					
**58**	m/3603	0.206	Non-biofilm producer	0.5	1	0.008	128	0.38	1	0	0	0	0					
**63**	m/3537	0.221	Non-biofilm producer	0.19	1	0.008	16	0.19	1	1	0	1	0					
**65**	6355m25	0.167	Non-biofilm producer	1	0.5	0.19	4	1.5	1	0	1	1	0					
**67**	g/3641	0.230	Non-biofilm producer	0.5	0.75	0.19	16	> 32	1	0	1	1	0					
**74***	MG1655	0.191	Non-biofilm producer	1	0.125	0.012	2	0.047	**1**	**0**	**0**	**1**	**0**					
**1**	5640m25	0.265	Weak biofilm producer	0.012	0.75	0.008	4	0.94	0	1	1	1	1					
**3**	5544m25	0.460	Weak biofilm producer	0.38	0.25	0.006	3	> 32	1	1	1	1	1					
**4**	5570m25	0.456	Weak biofilm producer	0.19	0.38	0.006	1.5	0.064	1	1	1	1	1					
**6**	5713m25	0.463	Weak biofilm producer	3	0.25	0.008	1	0.064	1	0	1	1	1					
**7**	5551m25	0.440	Weak biofilm producer	1	1	0.38	2	> 32	1	1	1	1	1					
**9**	5516m25	0.292	Weak biofilm producer	0.38	0.75	0.12	2	> 32	1	0	1	1	1					
**10**	5969m25	0.385	Weak biofilm producer	1.5	1	0.016	3	0.094	1	0	1	0	1					
**16**	5609m25	0.431	Weak biofilm producer	0.38	38	1	12	> 32	1	0	1	0	1					
**17**	5619m25	0.328	Weak biofilm producer	0.5	32	≤ 0.25	16	>16	1	0	1	1	1					
**18**	5678m25	0.418	Weak biofilm producer	0.5	0.75	> 1	128	≤ 8	1	0	1	1	1					
**26**	5768m25	0.431	Weak biofilm producer	3	0.75	0.008	>256	0.25	1	1	1	1	1					
**27**	5658m25	0.280	Weak biofilm producer	3	1	0.023	1.5	0.25	1	0	0	1	1					
**28**	5655m25	0.434	Weak biofilm producer	3	0.75	0.012	16	>16	0	0	1	1	0					

**44**	6434m25	0.466	Weak biofilm producer	1.5	1	> 32	32	> 32	1	0	1	1	0					
**45**	m/3699	0.395	Weak biofilm producer	3	2	0.19	>192	0.19	1	0	0	1	0					
**46**	m3622	0.390	Weak biofilm producer	2	0.5	0.012	3	0.25	1	1	1	1	0					
**48**	m/3795	0.333	Weak biofilm producer	2	0.75	0.008	>256	>16	1	0	1	1	0					
**54**	m/3533	0.403	Weak biofilm producer	3	0.38	0.023	>256	0.125	1	0	0	1	0					
**57**	6078m25	0.300	Weak biofilm producer	32	1	0.012	16	> 32	1	0	0	0	0					
**59**	g/3697	0.329	Weak biofilm producer	1.5	0.38	≤ 0.25	32	≤ 8	1	0	0	1	0					
**61**	6484m25	0.371	Weak biofilm producer	6	0.5	0.016	>256	> 32	1	0	1	1	0					
**62**	m/3697	0.251	Weak biofilm producer	1	0.75	0.016	16	0.094	1	0	0	1	0					
**64**	m3665	0.249	Weak biofilm producer	0.38	0.75	0.016	4	0.125	0	0	0	0	0					
**66**	M/3702	0.369	Weak biofilm producer	1	96	0.008	16	> 32	1	0	0	1	0					
**69**	m/3800	0.341	Weak biofilm producer	0.75	1	0.047	2	0.094	1	0	0	1	0					
**29**	3515	0.488	Moderate biofilm producer	1.5	0.75	0.016	16	0.25	0	0	1	1	0					
**5**	5735m25	0.609	Moderate biofilm producer	0.19	0.5	> 32	8	> 32	1	0	1	1	1					
**8**	5730m25	0.644	Moderate biofilm producer	0.5	0.75	0.008	0.094	1.5	1	0	1	0	1					
**15**	5549m25	0.686	Moderate biofilm producer	0.5	0.5	≤ 0.25	2	>16	1	0	0	1	1					
**19**	m3341	0.614	Moderate biofilm producer	1.5	1.5	0.012	8	0.25	1	0	0	1	0					
**21**	m338729	0.793	Moderate biofilm producer	0.5	0.5	0.008	8	0.19	1	0	1	1	1					
**22**	m3352	0.541	Moderate biofilm producer	0.75	1	0.008	16	0.125	1	1	1	1	1					
**23**	m/3541	0.637	Moderate biofilm producer	1.5	48	0.016	16	>16	1	0	1	1	1					
**24**	3463	0.733	Moderate biofilm producer	2	0.75	0.012	2	0.125	1	0	1	1	1					
**25**	5752m25	0.515	Moderate biofilm producer	3	0.75	0.023	3	0.125	1	0	1	0	0					
**31**	m/3632	0.582	Moderate biofilm producer	0.75	1	0.012	16	0.19	1	1	1	1	1					
**34**	m/3554	0.525	Moderate biofilm producer	6	1	> 32	8	> 32	1	0	1	1	1					
**39**	M3811 20.	0.676	Moderate biofilm producer	0.5	0.38	0.008	4	0.094	1	1	1	1	0					
**42**	6132m25	0.516	Moderate biofilm producer	0.5	> 256	8	8	> 32	1	0	0	1	1					
**49**	m/3787/25	0.601	Moderate biofilm producer	2	96	> 1	8	0.064	1	0	1	0	0					
**56**	M/3548	0.717	Moderate biofilm producer	4	0.5	0.012	4	1.5	1	0	0	1	0					
**60**	6022m25	0.622	Moderate biofilm producer	1	0.5	0.38	24	0.25	1	0	1	1	0					
**68**	m/3586	0.501	Moderate biofilm producer	1.5	0.5	0.008	2	1	1	0	0	1	0					
**70**	6469m25	0.529	Moderate biofilm producer	0.75	1	0.016	2	0.094	1	0	1	0	0					
**71**	6072m25	0.667	Moderate biofilm producer	0.75	0.75	0.125	8	0.25	1	1	1	1	0					
**73***	UPEC536	0.737	Moderate biofilm producer	0.38	0.5	0.012	1	0.125	1	1	1	1	0					
**2**	5465m	0.985	Strong biofilm producer	0.5	0.38	0.19	2	0.125	0	0	1	0	1	1.20E+10	8.50E+06	5.95E+06		
**13**	m337127	1.314	Strong biofilm producer	0.5	1.5	0.047	8	0.75	1	1	1	1	1	1.33E+10	1.60E+07	8.97E+06		
**20**	m3335	1.260	Strong biofilm producer	0.19	1.5	12	24	>32	0	0	1	1	1	1.40E+10	7.47E+07	6.23E+07	6.00E+05	1.20E+05

**32**	6046m25	1.146	Strong biofilm producer	>32	64	>32	8	>32	1	0	1	1	1	9.53E+10	1.17E+07	4.40E+06	2.53E+07	1.70E+07
**33**	6083m25	1.145	Strong biofilm producer	1.5	0.5	0.016	8	0.064	1	1	1	1	0	2.30E+10	9.33E+06	6.98E+06		
**35**	6130m25	1.726	Strong biofilm producer	48	0.75	0.016	2	0.125	1	0	0	0	0	5.80E+10	1.77E+07	1.13E+07		
**36**	M/3543	1.205	Strong biofilm producer	0.5	0.75	0.008	1	0.094	1	1	0	1	0	2.20E+10	1.73E+07	8.40E+06		
**37**	m/3541	1.311	Strong biofilm producer	0.5	48	0.012	6	>32	1	1	1	1	0	9.33E+09	7.10E+06	6.20E+06		
**38**	m/3714	1.192	Strong biofilm producer	48	64	>32	6	>32	1	0	0	0	0	2.85E+10	1.47E+07	6.13E+06	1.13E+07	5.42E+06
**40**	M3774 18)	1.680	Strong biofilm producer	0.5	0.75	0.016	1.5	0.064	1	0	1	0	0	1.40E+10	2.23E+07	1.73E+07		
**41**	6103m25	1.027	Strong biofilm producer	0.19	0.5	0.094	3	0.38	1	1	1	1	1	2.20E+10	5.30E+06	2.98E+06		
**43**	m3685	1.414	Strong biofilm producer	0.75	0.5	0.19	12	>32	1	1	1	1	1	2.22E+10	3.53E+07	7.67E+06		
**47**	M/3643	0.995	Strong biofilm producer	0.25	>128	>32	4	0.094	1	0	1	1	1	6.03E+10	4.03E+07	1.23E+08	9.90E+06	4.04E+06
**72***	CFT073	1.859	Strong biofilm producer	0.38	0.5	0.008	4	0.38	1	1	1	1	1	4.80E+10	8.60E+07	4.30E+07		

Table S2 Secretion system patterns across biofilm categories. Percentages are calculated as the proportion of n = 71 clinical isolates carrying each specific gene combination and are not mutually exclusive. T1 stands for *hlyA*, T2 – *gspD,* T5 – *ag43*, T6 – *hcp.* Counts represent co-detection (non-mutually exclusive); thus, isolates carrying all four markers contribute to all pairwise and triple combinations. None of the combinations is statistically significant after Benjamini-Hochberg false discovery rate FDR) correction.

Secretion system combinations	Strains with combination (n=71)	Non-Biofilm (n=13)	Weak (n=25)	Moderate (n=20)	Strong (n=13)	χ^2	p-value	FDR-adjusted q-value
T1+T2	21 (29.6%)	6	6	4	5	3.55	0.314	0.549
T1 + T5	23 (32.4%)	7	6	4	6	6.25	0.100	0.549
T1 + T6	14 (19.7%)	4	5	2	3	2.96	0.399	0.549
T2 + T5	43 (60.6%)	10	14	11	8	1.90	0.593	0.675
T2 + T6	30 (42.3%)	4	11	8	7	7.93	0.048	0.528
T5 + T6	29 (40.8%)	4	10	9	6	1.53	0.675	0.675
T1+T2+T5	21 (29.6%)	6	6	4	5	3.55	0.314	0.549
T1+T2+T6	14 (19.7%)	4	5	2	3	2.96	0.399	0.549
T1+T5+T6	14 (19.7%)	4	5	2	3	2.96	0.399	0.549
T2+T5+T6	26 (36.6%)	4	9	7	6	1.80	0.617	0.675
T1+T2+T5+T6	14 (19.7%)	4	5	2	3	2.96	0.399	0.549

Table S3 Associations between secretion-system marker-positive and marker-negative UPEC isolates. Values are reported as median MIC (µg/mL) for each group, with corresponding p-values (Mann-Whitney test with Benjamini-Hochberg FDR correction for multiple comparisons). Statistical significance is indicated as follows: ns, not significant; * adjusted p < 0.05.

Antibiotic	Gene marker	n	Median MIC gene (µg/mL)	MIC range (µg/mL)	adjusted p-value	Statistical significance
Ampicillin-sulbactam	*hlyA-*	48	8	0.094 – 256	0.452	ns
	hlyA+	23	4	1 – 256		
	gspD-	20	7	1 – 256	0.964	ns
	gspD+	51	8	0.094 – 256		
	ag43-	14	3.5	0.094 – 128	0.201	ns
	ag43+	57	8	1 – 256		
	hcp-	38	12	1 – 256	0.038	*
	hcp+	33	4	0.094 – 256		
Ciprofloxacin	*hlyA-*	48	0.023	0.008 – 32	0.018	*
	hlyA+	23	0.012	0.006 – 0.38		
	gspD-	20	0.016	0.008 – 32	1	ns
	gspD+	51	0.016	0.006 – 32		
	ag43-	14	0.016	0.008 – 32	1	ns
	ag43+	57	0.016	0.006 – 32		
	hcp-	38	0.016	0.008 – 32	1	ns
	hcp+	33	0.047	0.006 – 32		

Fosfomycin	*hlyA-*	48	1.5	0.19 – 48	0.038	*
	hlyA+	23	0.5	0.012 – 3		
	gspD-	20	1.5	0.19 – 48	0.121	ns
	gspD+	51	0.75	0.012 – 32		
	ag43-	14	1.125	0.38 – 48	0.332	ns
	ag43+	57	1	0.012 – 32		
	hcp-	38	1.5	0.19 – 48	0.018	*
	hcp+	33	0.5	0.012 – 32		
Gentamicin	*hlyA-*	48	0.75	0.25 – 256	0.474	ns
	hlyA+	23	0.75	0.25 – 48		
	gspD-	20	1	0.38 – 256	0.528	ns
	gspD+	51	0.75	0.25 – 128		
	ag43-	14	1	0.38 – 96	0.411	ns
	ag43+	57	0.75	0.25 – 256		
	hcp-	38	0.75	0.38 –96	0.883	ns
	hcp+	33	0.75	0.25 – 256		
Trimethoprim-sulfamethoxazole	*hlyA-*	48	1.25	0.064 – 32	0.586	ns
	hlyA+	23	0.25	0.047 – 32		
	gspD-	20	0.25	0.094 – 32	0.586	ns
	gspD+	51	0.38	0.047 – 32		
	ag43-	14	0.125	0.064 – 32	0.586	ns
	ag43+	57	0.75	0.047 – 32		
	hcp-	38	0.25	0.064 – 32	0.564	ns
	hcp+	33	1.5	0.047 – 32		

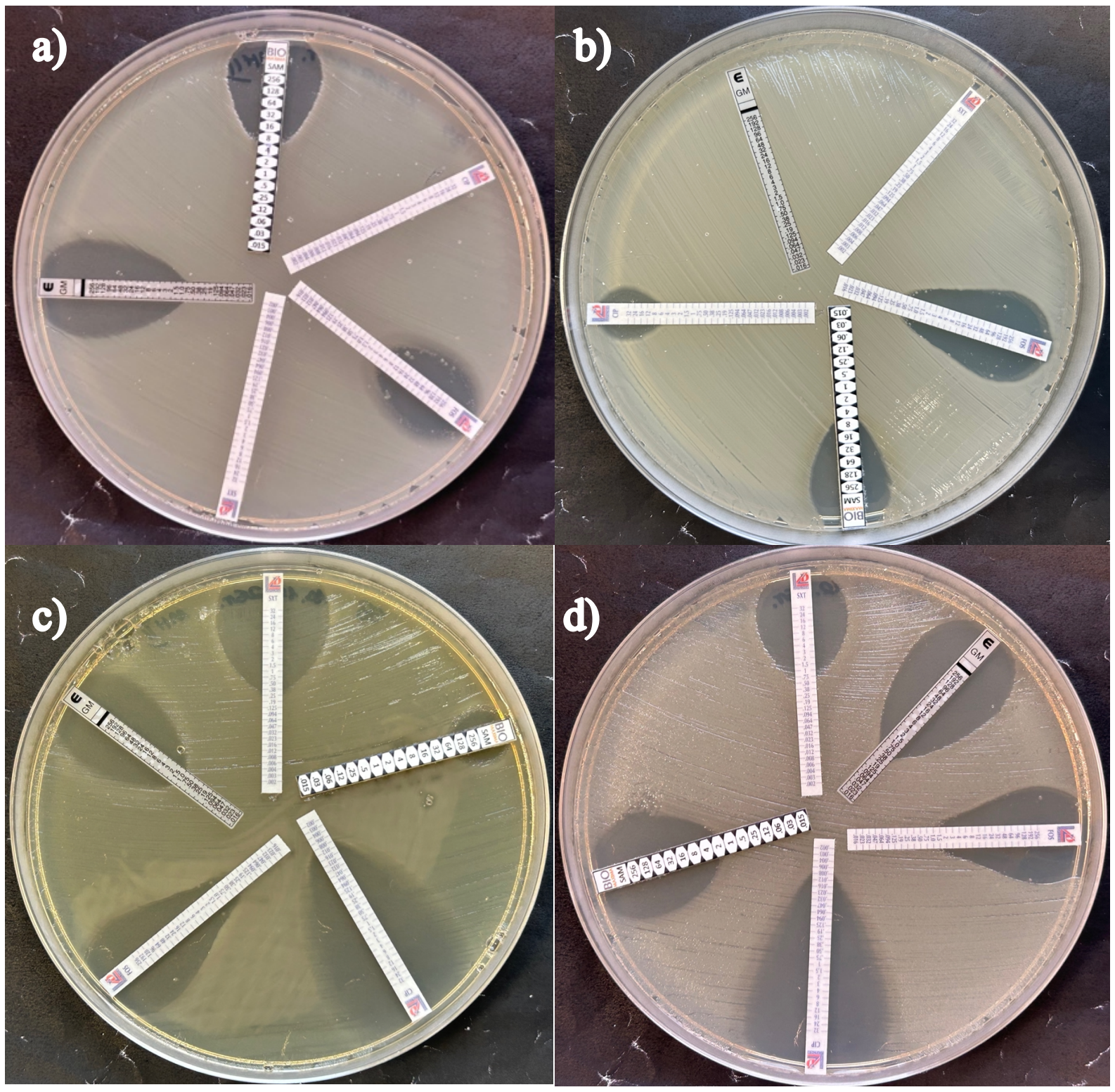

Figure S1 Representative Etest strips for antimicrobial susceptibility testing. Examples demonstrate: a) strain 34: CIP[R], TMP/SMX [R]; b) strain 42: CIP[R], TMP/SMX[R], GM[R]; c) strain 58: AMP[R]; d) strain 68: susceptible to all. Detailed data in Table S1.

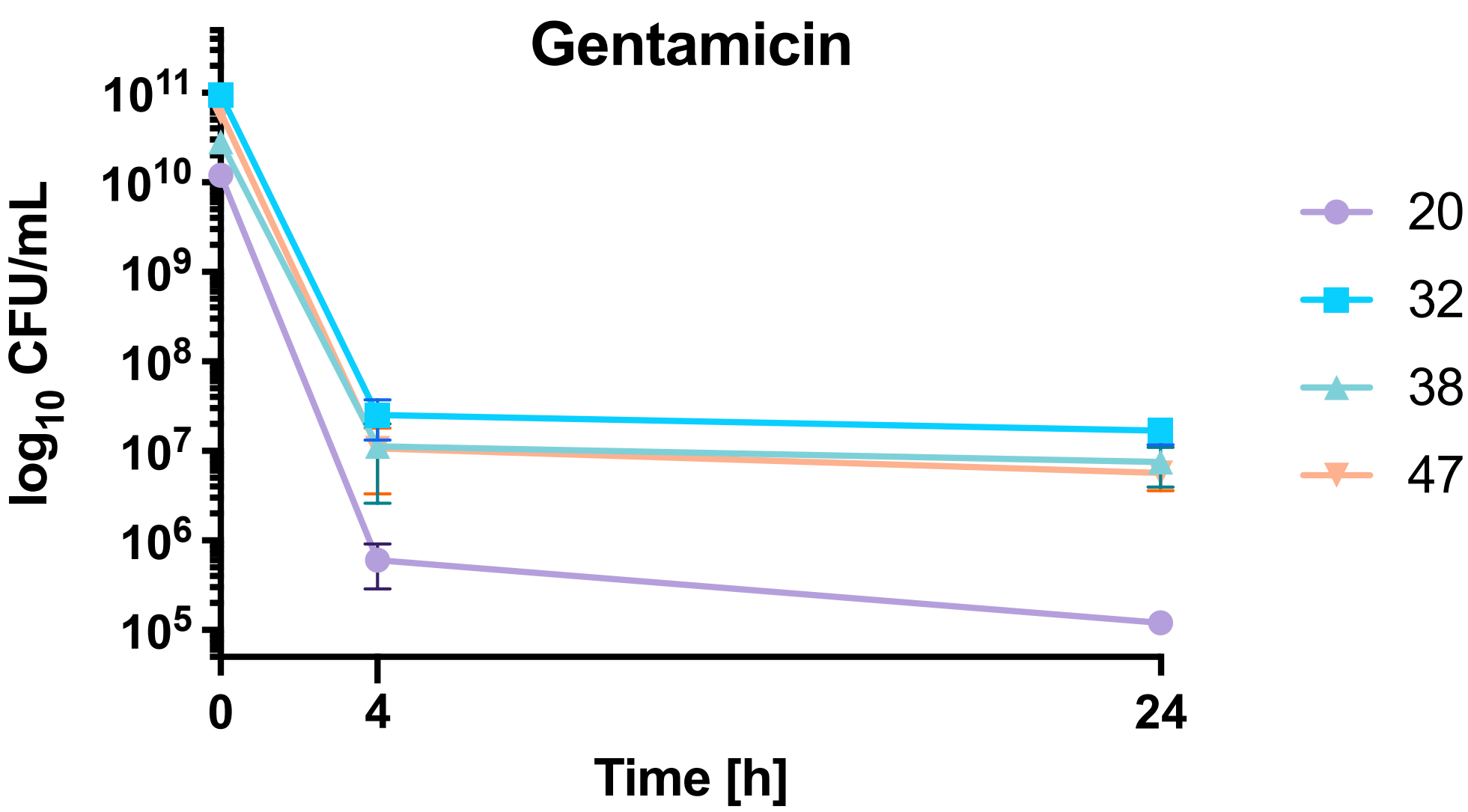

Figure S2 Persister-cell survival of ciprofloxacin-resistant, strong biofilm-producing UPEC isolates quantified by viable counts after gentamicin challenge. Bars indicate mean ± SD (n=3).

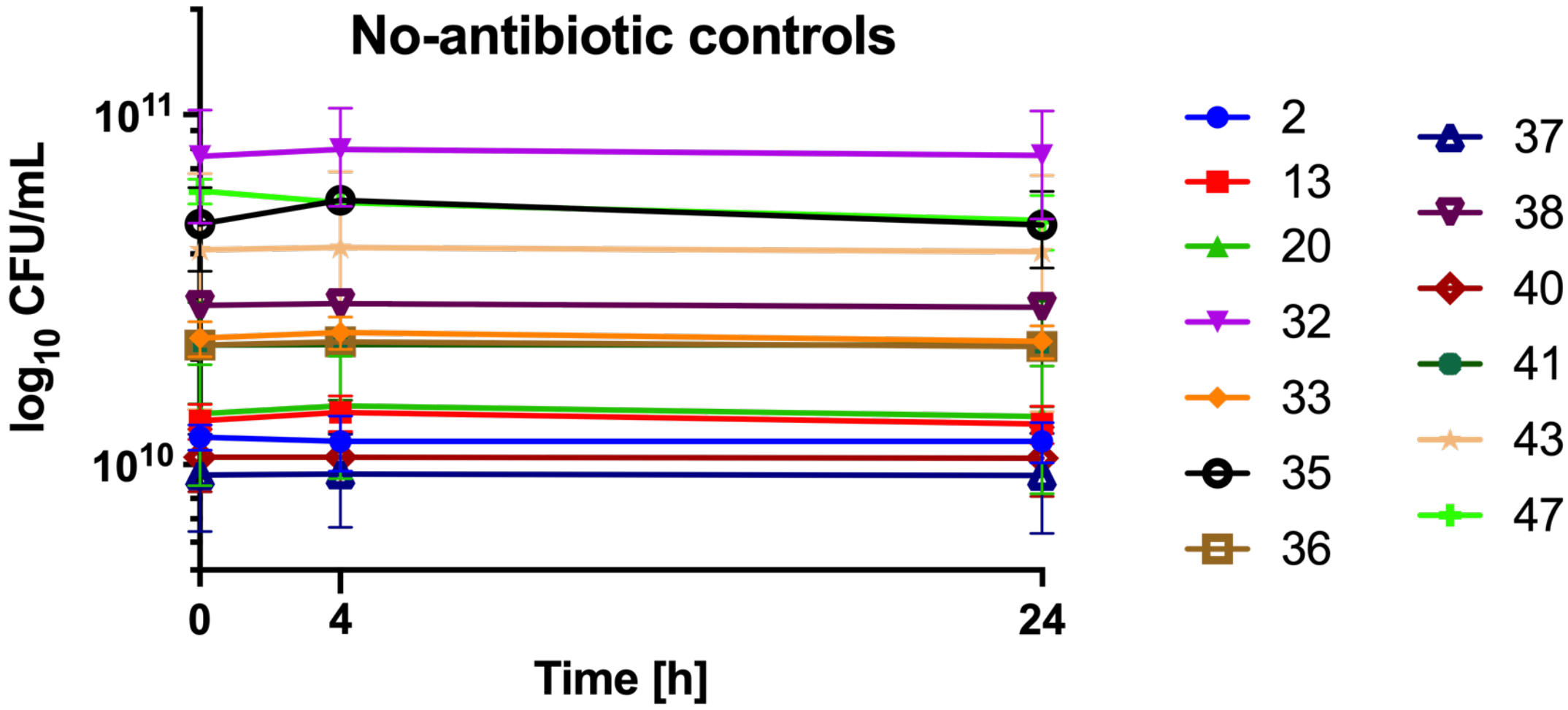

Figure S3 No-antibiotic biofilm controls used in the biofilm persister assay. Untreated biofilms were processed using the same biofilm disruption and CFU enumeration procedure as antibiotics treated groups. Data indicate mean ± SD (n=3).

Table S4 Oligonucleotide primers used in this study and expected amplicon sizes. Primer sequences are shown 5′-3′, and expected PCR product lengths are given in base pairs (bp).

Primer name	Secretion System	Sequence (5'-3')	Product size
hly_For	I	GTCTGCAAAGCAATCCGCTGCAAATAAA	561 bp
hly_Rev		CTGTGTCCACGAGTTGGTTGATTAG	
gsp_For	II	ATTCGCCGTGCTCAGGTG	799 bp
gsp_Rev		GTCGGACGGATAAACACCATCAGG	
ag43_For	V	CTGGAAACCGGTCTGCCCTT	433 bp
ag43_Rev		CCTGAACGCCCAGGGTGATA	
hcp_For	VI	GCGGATATCAAAGGTTCCGTGGAC	400 bp
hcp_Rev		CTTTGTAAGTCCAGGTGATTTTCTCGT	
fimH_For	-	CTGCCGTGAATGTGGGGC	600 bp
fimH_Rev		TCAACTGTACGCCGACGC	

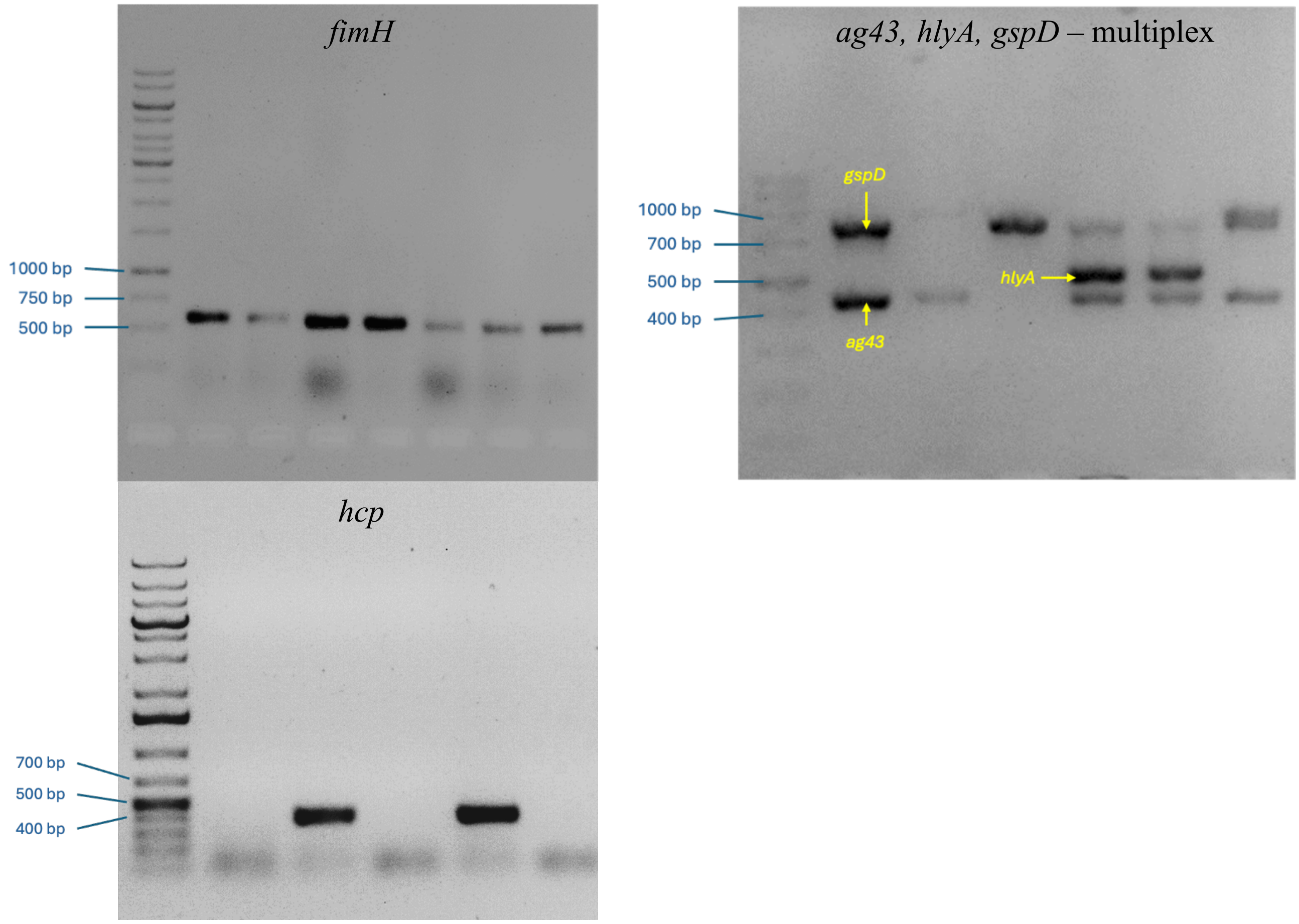

Figure S4 Representative agarose gel electrophoresis images of PCR amplicons for *fimH*, *ag43, hlyA, gspD* in multiplex PCR (upper panels), and *hcp* (lower panel). As DNA molecular weight marker a GeneRuler 1 kb DNA Ladder (Thermo Scientific™) was used for the *fimH* and a GeneRuler 1 kb Plus DNA Ladder (Thermo Scientific™) was used for the multiplex gels and *hcp* gels.
